# Access to Naloxone and Preferences for Its Online Purchase Among Individuals Affected by Opioid Misuse

**DOI:** 10.7759/cureus.91238

**Published:** 2025-08-29

**Authors:** Rahul Garg, James Lott

**Affiliations:** 1 Division of Institutional Effectiveness, Alabama College of Osteopathic Medicine, Dothan, USA; 2 Department of Pharmacy, Scripted, Chicago, USA

**Keywords:** drug addiction, naloxone, online pharmacy, opioid, pharmacist

## Abstract

Introduction

Naloxone is a lifesaving medication that can prevent deaths in opioid overdose situations. However, barriers to access and purchasing naloxone still exist. Online pharmacies present an important venue to expand access to naloxone. We examined the access to naloxone, counseling for opioid overdose prevention drugs, and preferences regarding online purchase of naloxone among individuals affected by opioid misuse.

Methods

We conducted an online survey among 504 individuals who were either personally affected or had family members/friends who were affected by the misuse of opioids.

Results

The study included participants from all 50 US states, among whom 37.5% were personally affected and 30.1% had a family/friend who was affected by opioid misuse. Although 40.3% were current or past users of heroin, and 91.1% of participants or their loved ones had taken prescription opioids, a majority (72.5%) had never purchased or been given a drug to prevent an opioid overdose. The most common barriers to purchasing naloxone included cost (60.5%), inability to get a prescription (30.9%), and unavailability at pharmacies (30.9%). About half of the participants (46.4%) indicated online pharmacy as their first choice for purchasing naloxone. Factors significantly associated with preferring online purchase of naloxone included not being comfortable with talking to a doctor/pharmacist regarding opioid use and previous experience of issues with purchasing naloxone.

Conclusions

Many individuals who might need naloxone face barriers to getting naloxone from a pharmacy. Availability of naloxone from online pharmacies and higher insurance coverage can significantly increase access and potentially reduce opioid overdose deaths.

## Introduction

Overdose deaths involving any opioid dramatically increased over the past decade from 21,089 in 2010 to 68,630 in 2020 in the US [1]. Some of the national efforts to prevent opioid overdoses include better data surveillance, stringent opioid drug prescription guidelines [2], a 64.4% increase in the use of prescription drug data monitoring programs in 2019 [3], and a 106% increase in the number of practitioners certified to administer buprenorphine-based opioid addiction treatment from 2014 to 2019 in the US [4].

Due to these efforts, from 2020 to 2021, deaths due to prescription opioids without synthetic opioids other than methadone decreased from 7,790 to 7,062, and those due to heroin decreased from 13,165 to 9,173 [5]. Further, deaths involving synthetic opioids other than methadone (primarily illicitly manufactured fentanyl) decreased from 73,838 overdose deaths in 2022 to 72,776 in 2023 [1]. Overall opioid-involved overdose deaths rose from 49,860 in 2019 to 81,806 in 2022 but significantly decreased to 79,358 in 2023 [1]. The evolving complexity and urgency of the opioid epidemic call for continued investigation and a more tailored approach to combat the crisis.

The Centers for Disease Control and Prevention strongly recommends an increased use of naloxone to curb fentanyl-related overdose deaths [5]. Naloxone is an opioid antagonist that is used to reverse respiratory depression and prevent death during opioid overdose situations [6]. In the US, all 50 states and the District of Columbia have enacted naloxone access laws (NALs) to increase the dispensing and use of naloxone. Some of the NALs include statewide standing prescription orders for pharmacists to prescribe naloxone, prescription of naloxone to a third-party individual who can potentially help someone with an overdose (Good Samaritan law), and co-prescribing of naloxone with high-risk opioid prescriptions (high opioid strength or dose) [7]. The NALs led to a doubling of naloxone prescriptions from 2017 to 2018 [7] and a reduction in prescription overdose deaths [8].

Despite policy changes, several access barriers still exist, and naloxone is under-prescribed and underutilized for patients at a high risk of overdose [9]. Common patient- and provider-level barriers to naloxone use include patient stigma, experiences and beliefs regarding the treatment, cost and availability of naloxone at the pharmacy store, and low provider knowledge and readiness regarding the dispensing of naloxone [10]. Although retail pharmacies are permitted to dispense naloxone without a prescription, the availability and actual dispensing of naloxone remain low in several US states [11]. Despite higher rates of overdose deaths, pharmacies in rural counties [12] and in neighborhoods with greater poverty [13] were less likely to sell naloxone.

Less than optimal availability of naloxone in brick-and-mortar pharmacies is further compounded by pharmacists’ lack of willingness to offer and dispense naloxone [14]. Several factors affect pharmacists’ readiness to dispense naloxone [15], including lack of knowledge regarding the standing prescription orders [16], feeling uncomfortable with dispensing naloxone without a prescription [17], lack of training [14,17], stigma about opioid use disorder (OUD) [11], time constraints [11,14,18], and concerns about the type of clientele who would frequent the pharmacy [18]. Another significant barrier to the purchase of naloxone is the cost of prescription [11,16,18]. In 2018, out-of-pocket costs were required for about 57.7% of naloxone prescriptions, with amounts of <$10 (24.5%), $10.01-$50 (21.9%), and >$50 (5.8%) [9]. Individuals without health insurance had the highest out-of-pocket costs, with 31.0% of individuals paying >$50 in 2018.

In addition to cost, patients affected by opioid misuse might face other barriers at individual and contextual levels that are currently unexplored. One study conducted among naloxone purchasers with unknown opioid use status found that perceived stigma from pharmacists and previous experiences at the pharmacy can affect the likelihood of obtaining naloxone [19]. Even though awareness of naloxone is high among patients with opioid misuse or addiction [20], access to naloxone and the actual possession and use of naloxone are low [21]. Access to naloxone and preferences regarding the purchase of naloxone warrant further investigation from the patients’ perspective. Limited studies have explored the barriers faced by individuals who currently use drugs and other individuals who are affected by opioid misuse [22,23].

Recently, naloxone has become available through online pharmacies and websites that assist with the standing orders for naloxone prescriptions, provide telehealth for naloxone, and connect patients with pharmacies dispensing naloxone [24]. In addition to pharmacies and healthcare clinics, harm reduction organizations, universities, and some states also provide free and low-cost naloxone through clinics, mail, vending machines, and libraries [25,26].

Given the existing barriers with naloxone dispensed through pharmacy stores and provider-related stigma, online pharmacy presents an important, previously unexplored venue to expand access to naloxone. We could not find a previous study on patients’ preferences regarding purchasing naloxone through an online pharmacy. The objective of this study was to examine access to naloxone, access to information regarding opioid overdose reversal drugs, and the comparison of online pharmacy vs. traditional access preferences to naloxone among individuals affected by opioid misuse.

## Materials and methods

Eligibility and recruitment

We conducted an online cross-sectional survey to collect data at Chicago State University from June to August 2023 by using SurveyMonkey. The study was approved by the Chicago State University Institutional Review Board (protocol number 039-12-20). To reduce opioid overdose deaths, all US states and the District of Columbia have passed laws to allow the purchase of naloxone by both opioid users and lay responders who are in a position to help their friend or family member in an opioid overdose situation [7]. Hence, we included individuals above 18 years of age who were either personally affected or had a family member or friend who was affected by opioid misuse to measure access and preferences regarding naloxone purchase. The survey link was posted on social media support groups for individuals affected by opioids on Facebook and Reddit. The support groups included members who were current users of opioids, addicted to or dependent on opioids, or had family members or friends who were affected by misuse of opioids. The survey participation was voluntary and completely anonymous. We did not provide any remuneration or incentive to participate in the study. Only those participants who read and accepted the consent form were allowed to take the survey. A total of 525 individuals participated in the study. We excluded 15 participants because they were neither personally affected nor did they know a family member or friend who was affected by opioids. Further, SurveyMonkey records participants’ IP addresses with each survey response. We deleted six responses that had duplicate IP addresses, which resulted in the final sample size of 504.

Survey measures

We designed the survey measures by reviewing similar studies with validated surveys in the literature [27-29]. Further, we conducted face validity of the survey measures by beta-testing the survey with two university students who had purchased naloxone in the past.

Demographic Characteristics

Participants’ demographic characteristics included state and region of residence (Table 1).

**Table 1:** Study participants’ state of residence

State	n	%	State	n	%
Alabama	10	2.3	Missouri	5	1.2
Alaska	4	0.9	Nebraska	1	0.2
Arizona	7	1.6	Nevada	5	1.2
Arkansas	3	0.7	New Hampshire	1	0.2
California	29	6.7	New Jersey	13	3
Colorado	7	1.6	New Mexico	4	0.9
Connecticut	6	1.4	New York	32	7.4
Delaware	3	0.7	North Carolina	12	2.8
Florida	19	4.4	North Dakota	1	0.2
Georgia	8	1.8	Ohio	26	6
Hawaii	1	0.2	Oklahoma	4	0.9
Idaho	4	0.9	Oregon	4	0.9
Illinois	11	2.5	Pennsylvania	29	6.7
Indiana	14	3.2	Rhode Island	2	0.5
Iowa	3	0.7	South Carolina	10	2.3
Kansas	2	0.5	Tennessee	9	2.1
Kentucky	7	1.6	Texas	29	6.7
Louisiana	4	0.9	Utah	4	0.9
Maine	5	1.2	Vermont	2	0.5
Maryland	7	1.6	Virginia	10	2.3
Massachusetts	15	3.5	Washington	19	4.4
Michigan	19	4.4	West Virginia	3	0.7
Minnesota	10	2.3	Wisconsin	8	1.8
Mississippi	1	0.2	Wyoming	3	0.7

Use, Misuse, and Sources of Opioid Drugs

The use, misuse, and sources of opioids were measured by the following five questions: (1) Who is affected by opioids? (self, my family, my friend, or other (please specify)); (2) Do you (or a loved one) take or have taken prescription opioids (e.g., Lortab, Percocet, Oxy, oxycodone, fentanyl, codeine, and purple syrup)? (yes/no); (3) Do you know a family member or friend who has misused prescription opioids? (yes/no); (4) Do you use or have you used heroin? (yes/no); (5) How do you get your prescription opioids or heroin? (from a doctor with a prescription, online (dark web), from friends, at a street market, or other (please specify)).

Access to Opioid Overdose Prevention Drugs

We provided a brief description of naloxone to the participants before asking questions regarding access to opioid overdose prevention drugs: “Naloxone is a drug that blocks the effects of opiates and reverses the effects of opioids, including respiratory depression, sedation, and hypotension. NARCAN® Nasal Spray is a prescription medicine used for the treatment of an opioid emergency, such as an overdose or a possible opioid overdose with signs of breathing problems and severe sleepiness or not being able to respond. The participants’ experiences and preferences regarding the purchase of drugs to prevent opioid overdose were measured by the following four questions: (1) Have you ever purchased or been given a drug that can prevent overdose? (yes/no); (2) Would you be willing to purchase Naloxone/Narcan or other overdose reversal drugs? (yes/no); (3) Have you tried to purchase or thought about purchasing naloxone/Narcan, but experienced issues getting it, or decided you no longer wanted to purchase it? (yes/no); (4) What issues did you run into? (could not get prescription from doctor or pharmacist, product out of stock, pharmacy does not carry, too expensive, or no pharmacy access). Our primary outcome of preferences regarding online purchase of naloxone was measured by the following question: Naloxone (an opioid overdose reversal drug) is now available without a prescription in many states. How would you prefer to purchase naloxone? (Please rank in order of preference: pharmacy, online, or other).

Access to Information Regarding Opioid Overdose Prevention

We utilized the following three questions to measure participants’ access to information regarding drugs to treat or prevent opioid overdoses: (1) Has anyone ever discussed with you a drug that can prevent opioid overdoses? (yes/no); (2) Based on your experience with prescription opioid and heroin use, how comfortable are you talking with your doctor or pharmacist about opioid use? (not at all, slightly, moderately, very, or extremely); (3) Where do you go to seek information about addiction treatment services and options? (online, by asking a medical professional (doctor, pharmacist, or counselor), or other (please specify)).

Statistical analysis

Our study analyzed the preference to purchase naloxone online among those with personal experience with opioid misuse as compared to those without personal experience (only have family/friends affected by opioid misuse). We classified the participants into three groups: those who only personally use opioids, those who do not use opioids themselves (family/friend only), and those with both personal experience and know a family/friend with opioid misuse. We compared the participants for ranking online pharmacy as their first choice for purchasing naloxone, as compared to a pharmacy or other sources, with chi-square tests. We also used chi-square tests to analyze factors associated with ever purchasing or being given a drug that can prevent overdose. Fisher’s exact test was used for bivariate analysis with low cell counts. We used logistic regression analysis for the multivariate adjusted analysis of factors associated with ranking online purchase of naloxone as the first choice. We adjusted the logistic regression analysis for individuals affected by opioids, source of opioids, use of heroin, having a family member or friend who misused prescription opioids, access to information regarding opioid overdose drugs, and experience with purchasing opioid overdose drugs. All data analyses were conducted using the SAS 9.4 software (SAS Institute Inc., Cary, NC, USA) [30].

## Results

Participants’ characteristics

**Figure 1:**
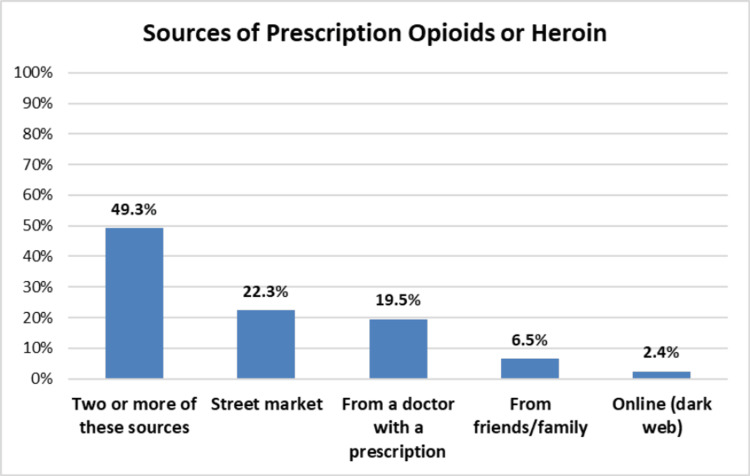
Participants’ sources of prescription opioids or heroin

**Figure 2:**
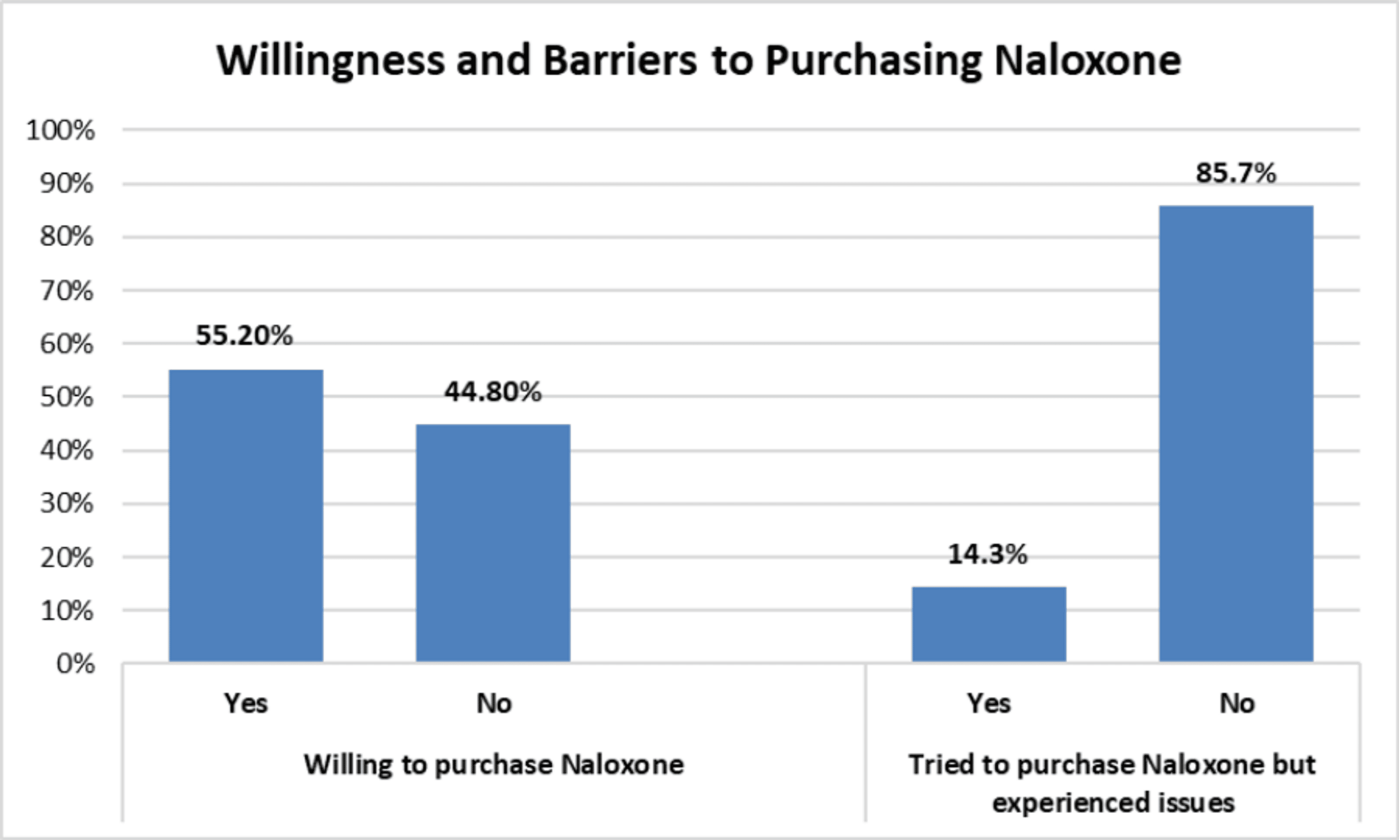
Participants’ willingness and barriers toward purchasing naloxone

Participants’ characteristics are listed in Figure 1, Figure 2, and Table 1. The study included participants from all regions and states of the US, with a higher proportion of individuals from the South and Northeast regions (Table 1). About 30.4% of the participants were both personally affected and had a family or friend who was affected by opioid use disorder (OUD; Table 2).

**Table 2:** Bivariate chi-square analysis of factors associated with participants’ preference to purchase naloxone online ^*^ Statistically significant at p < 0.05 from chi-square tests

Variable	Total, n (%)	Prefer to purchase naloxone online		Chi-square test statistic value	p-Value
	504	Yes, 155 (46.4%)	No, 179 (53.6%)		
Region				0.92	0.822
Northeast	105 (24.1%)	34 (50.0%)	34 (50.0%)		
Midwest	100 (23.0%)	27 (42.2%)	37 (57.8%)		
South	139 (32.0%)	45 (47.4%)	50 (52.6%)		
West	91 (20.9%)	25 (44.6%)	31 (55.4%)		
Individual affected by opioids				2.73	0.256
Self	189 (37.5%)	60 (46.9%)	68 (53.1%)		
Family/friend only	162 (32.1%)	45 (52.9%)	40 (47.1%)		
Self and family/friend	153 (30.4%)	50 (41.3%)	71 (58.7%)		
Source of prescription opioids or heroin^*^				10.35	0.035
From a doctor with a prescription	84 (19.5%)	35 (61.4%)	22 (38.6%)		
Street market	96 (22.3%)	23 (33.8%)	45 (66.2%)		
From friends/family	28 (6.5%)	8 (42.1%)	11 (57.9%)		
Online (dark web)	10 (2.4%)	6 (60.0%)	4 (40.0%)		
Two or more of these sources	212 (49.3%)	73 (47.1%)	82 (52.9%)		
Current or past use of heroin				3.79	0.061
Yes	203 (40.3%)	64 (40.8%)	93 (59.2%)		
No	301 (59.7%)	91 (51.4%)	86 (48.6%)		
Do you (or a loved one) take or have taken prescription opioids?				0.62	0.502
Yes	459 (91.1%)	147 (47.0%)	166 (53.0%)		
No	45 (8.9%)	8 (38.1%)	13 (61.9%)		
Know a family member or friend who misused prescription opioids				0	1
Yes	38 (7.5%)	13 (46.4%)	15 (53.6%)		
No	466 (92.5%)	142 (46.4%)	164 (53.6%)		
Anyone ever discussed a drug to prevent opioid overdose				1.72	0.223
Yes	309 (61.3%)	106 (44.2%)	134 (55.8%)		
No	195 (38.7%)	49 (52.1%)	45 (47.9%)		
Comfortable with talking to a doctor or pharmacist regarding opioid use^*^				4.65	0.032
Not at all^*^	165 (37.8%)	68 (54.0%)	58 (46.0%)		
Slightly to extremely comfortable	272 (62.2%)	87 (41.8%)	121 (58.2%)		
Source of information for addiction treatment options and services				0.2	0.978
Online	74 (63.8%)	27 (42.2%)	37 (57.8%)		
Medical professional (doctor, pharmacist, or counselor)	28 (24.1%)	12 (46.2%)	14 (53.8%)		
Both online and medical professionals	2 (1.7%)	1 (50.0%)	1 (50.0%)		
Other sources	12 (10.4%)	4 (40.0%)	6 (60.0%)		
Ever purchased or given a drug that can prevent opioid overdose				1.44	0.23
Yes	125 (24.8%)	45 (41.7%)	63 (58.3%)		
No	379 (75.2%)	63 (58.3%)	116 (51.3%)		
Willing to purchase naloxone or other drugs that can prevent opioid overdose				0.04	0.906
Yes	278 (55.2%)	105 (46.0%)	123 (54.0%)		
No	226 (44.8%)	50 (47.2%)	56 (52.8%)		
Tried to purchase but experienced issues in getting naloxone^*^				4.21	0.043
Yes	72 (14.3%)	34 (58.6%)	24 (41.4%)		
No	432 (85.7%)	121 (43.8%)	155 (56.2%)		

Participants’ use of opioids

Most participants (91.1%) were either past or current users of prescription opioids, and 40.3% were past or current users of heroin (Table 2). Only about 7% knew a family member or friend who had misused prescription opioids. Street market was the most common source of opioids, with almost half of the participants (49.3%) obtaining opioids from multiple sources (Table 2).

Counseling and information about opioid overdose treatment

About 39.0% of participants indicated that no one had ever discussed a drug to prevent opioid overdose with them (Table 2). A large percentage of participants were not at all comfortable (37.8%) or slightly comfortable (15.5%) talking to a doctor or pharmacist regarding opioid use, with others being moderately (16.2%), very (14.9%), or extremely (15.6%) comfortable. Online websites were the most common source of information (63.8%) for addiction treatment options and services, followed by a medical professional (doctor, pharmacist, or counselor; 24.1%) (Table 2).

Purchase of opioid overdose prevention drugs

Despite all participants either being personally affected or having a friend/family member affected by OUD, a majority of the study participants (75.2%) had never purchased or been given a drug to prevent opioid overdose (Table 2). From the chi-square test, a higher percentage of participants who were both personally affected and had a family/friend who was affected (33.3%) had purchased or were given a drug to prevent opioid overdose as compared to individuals who were personally affected (18.5%; p = 0.008). A lower percentage of individuals who were not at all comfortable with talking to a doctor or pharmacist regarding opioid use (32.0%) had ever purchased or been given a drug to prevent overdose. Further, a higher percentage of individuals who were past or current users of heroin (44.8%; p < 0.0001) and those who knew a family member or friend who had misused prescription opioids (39.5%; p = 0.029) had ever purchased or been given a drug to prevent overdose. About 55.2% of participants were willing to purchase naloxone or other drugs to prevent opioid overdose, and 14.3% of participants had tried to purchase or thought about purchasing naloxone/Narcan in the past but experienced issues getting it. The most common barriers faced with purchasing naloxone among those who tried to purchase naloxone (81 participants) included cost (60.5%), inability to get a prescription from a doctor or pharmacist (30.9%), unavailability at the pharmacy store (30.9%), lack of access to a pharmacy (13.6%), and naloxone being out of stock (6.2%). About half of the participants (47.3%) ranked in-store pharmacy as the first preference for purchasing naloxone, with the other half indicating online pharmacy (46.4%) and other sources (6.3%) as their first preference.

Factors associated with preference to purchase naloxone online

From logistic regression analysis, individuals who were not at all comfortable with talking to a doctor or pharmacist regarding opioid use (adjusted OR (95% CI): 1.70 (1.02, 2.82)) and those who faced issues in getting naloxone (2.61 (1.37, 4.98)) were significantly more likely to prefer online pharmacy for purchasing naloxone (Table 3). Further, individuals who obtained opioids illegally from the streets (0.31 (0.14, 0.68)) were less likely to prefer an online pharmacy for purchasing naloxone as compared to those who obtained it from a doctor with a prescription. From logistic regression analysis, the preference to buy naloxone online did not differ between individuals who were personally affected or had family/friends who were affected by opioids.

**Table 3:** Adjusted ORs for the factors adjusted for multivariate logistic analysis of participants’ preference to purchase naloxone online ^*^ Statistically significant at p < 0.05 Ref. = reference category The table lists the variables that were adjusted for the logistic multivariate analysis.

Factors	Adjusted OR	95% CI	Wald chi-square statistics	p-Value
Individual affected by opioids				
Family/friend only	1.09	0.59, 2.02	0.08	0.779
Self and family/friend	0.83	0.47, 1.46	0.42	0.516
Self	Ref.			
Source of prescription opioids or heroin				
Street market^*^	0.31	0.14, 0.68	8.41	0.004
From friends/family	0.4	0.13, 1.20	2.69	0.101
Online (dark web)	0.21	0.03, 1.58	2.3	0.13
Two or more of these sources	0.57	0.28, 1.12	2.66	0.103
From a doctor with a prescription	Ref.			
Current or past use of heroin				
Yes	0.76	0.44, 1.33	0.91	0.34
No	Ref.			
Know a family member or friend who misused prescription opioids				
Yes	6.31	0.98, 40.41	3.78	0.052
No	Ref.			
Anyone ever discussed a drug to prevent opioid overdose				
Yes	0.84	0.47, 1.51	0.34	0.557
No	Ref.			
Comfortable with talking to a doctor or pharmacist regarding opioid use				
Not at all^*^	1.7	1.02, 2.82	4.19	0.041
Slightly to extremely comfortable	Ref.			
Experienced issues in getting naloxone				
Yes^*^	2.61	1.37, 4.98	8.44	0.004
No	Ref.			
Ever purchased or given a drug that can prevent opioid overdose				
Yes	0.88	0.48, 1.63	0.15	0.695
No	Ref.			
Willing to purchase naloxone or other drugs that can prevent opioid overdose				
Yes	1.01	0.60, 1.70	0	0.978
No	Ref.			

## Discussion

Our study investigated access to naloxone and factors associated with preferring online purchase of naloxone from the perspective of individuals affected by opioid misuse. We found that the street market was the largest source of opioids as compared to a doctor with a prescription or friends and family. Even though all study participants were affected by opioid misuse and over half of them were willing to purchase naloxone, a majority had never purchased or been given an opioid overdose reversal drug. We found that high cost, unavailability at the store, and provider stigma related to naloxone were the main barriers for obtaining naloxone through pharmacy stores [19].

We found that individuals who had experienced issues purchasing naloxone in the past were more likely, and those who obtained opioids illegally from the streets were less likely, to prefer an online pharmacy for naloxone. Individuals who use opioids, especially those acquiring opioids illicitly, often face homelessness, poverty, lack of insurance, and limited internet access, which can make it difficult to use online pharmacies [31]. These structural barriers hinder both awareness and the ability to obtain naloxone online or in person. Online pharmacies also face the challenge of insurance coverage and might be costlier to obtain naloxone from as compared to a physical pharmacy [32]. Increased availability, awareness, and insurance for naloxone through online pharmacy stores will likely increase the purchase and use of naloxone in high-risk situations.

We found that over half of the participants were not at all or slightly comfortable talking to a doctor or pharmacist regarding opioid use. Pharmacists play a significant role in every aspect of care for patients at risk of opioid poisoning. Increased training of pharmacists in the use and dispensing of naloxone and patient education regarding naloxone holds great promise in reducing provider stigma among individuals in need of naloxone. Pharmacist-led programs to increase naloxone prescribing, such as co-prescribing with high-risk opioid therapy [33] and provider education regarding naloxone standing orders [34], have proven successful in primary care. Increased availability of naloxone through online pharmacies will further help individuals who are not comfortable with talking to a provider regarding drug use or overdose reversal drugs. Online pharmacies also make it easier to prescribe naloxone by utilizing standing prescription orders and connecting patients with pharmacies that dispense naloxone.

We found cost to be a significant access barrier, and increased insurance coverage of naloxone and a reduction in the out-of-pocket costs can dramatically increase the access and use of naloxone. The impact of increased coverage is exemplified by Medicaid expansion, which led to a nearly 10% increase in dispensing of naloxone [35]. There is an urgent need to further increase the availability and dispensing of naloxone across the nation. Mandating the availability of naloxone at all pharmacies across the US might improve the availability and use of naloxone in overdose situations. An increasing number of online pharmacies are providing naloxone without the need for a prescription. Although online dispensing of naloxone will increase access to naloxone, the insurance coverage and affordability may vary depending on the type of product and insurance plan [32]. All states provide coverage for naloxone as an outpatient prescription drug under the Medicaid prescription drug benefit. Further, under federal regulations, most private plans are required to provide coverage for naloxone as part of the Essential Health Benefits program. Many state-regulated health plans also require coverage of naloxone [32]. Continued insurance coverage of naloxone dispensed by online pharmacies is required to improve access to naloxone.

Converting naloxone to over-the-counter status, as done in certain countries like Italy and Australia, improved access and led to an increase in sales and use of naloxone in these countries [36]. The US FDA recently approved one product of naloxone to be an over-the-counter drug, which is a welcome step to increase accessibility to naloxone [37]. However, over-the-counter naloxone may not be covered by all health coverage programs, and the retail price could be unaffordable for many individuals. All states must ensure continuous coverage of over-the-counter and online dispensing of naloxone by Medicaid and private insurance plans to ensure affordability of this lifesaving medication.

In addition to pharmacies and healthcare clinics, harm reduction organizations, universities, and some states also provide free and low-cost naloxone through clinics, mail, vending machines, and libraries. Through these services, anyone can obtain a naloxone kit for emergency use. Such programs can be highly influential in creating awareness, reducing stigma regarding naloxone, and increasing the use of naloxone in emergency overdose situations. Such naloxone distribution programs must be accompanied by education regarding opioid overdose identification and appropriate use of naloxone in overdose situations. While increased availability of naloxone is necessary to reduce overdose deaths, there is a lack of standardized or consistent data collection across states on how naloxone distribution programs affect opioid mortality rates. The long-term impact of such distributions on actual drug-use behavior and overall opioid overdose deaths is warranted.

Our study had some limitations. The study included self-reported use and misuse of prescription and illicit opioids. Due to using an online survey, our study may have only selected participants who have access to the internet and are more comfortable with using online platforms. Such individuals are potentially more likely to be favorable toward online pharmacies. Study participants included individuals who were personally affected or had a family member or friend who was affected by opioids. However, our study did not find differences in preferences to purchase naloxone online by the type of participant. The study did not measure age and sex demographic information of the participants due to data privacy and to increase study response rates. Hence, we could not analyze racial differences in access to naloxone and preferences to purchase naloxone online. The strengths of the study include a large survey sample size with participants from all regions and states of the US. This is the first study to measure access barriers and preferences regarding online purchase of naloxone directly among individuals affected by opioid misuse. Our study explored the potential of online pharmacies as a unique and upcoming source of naloxone to reduce access barriers among individuals affected by opioids.

## Conclusions

The current study examined access to naloxone, counseling for opioid overdose prevention drugs, and preferences regarding online purchase of naloxone among a national sample of individuals affected by opioid misuse. People who previously faced difficulties buying naloxone are more likely, while those obtaining opioids illicitly are less likely, to prefer online pharmacies. Cost, limited insurance coverage, and provider stigma significantly restrict naloxone access, but pharmacist training, co-prescribing programs, and patient education can help reduce these barriers. The findings of this study are relevant to policymakers for reducing the cost of naloxone and improving the insurance coverage of over-the-counter and online naloxone purchases.

## References

[1] (2025). Drug overdose deaths: facts and figures. https://nida.nih.gov/research-topics/trends-statistics/overdose-death-rates.

[2] (2025). Understanding the opioid overdose epidemic. https://www.cdc.gov/overdose-prevention/about/understanding-the-opioid-overdose-epidemic.html.

[3] American Medical Association (2025). Physicians’ Progress Toward Ending the Nation’s Drug Overdose and Death Epidemic. Opioid Task Force.

[4] (2025). Early Changes in Waivered Clinicians and Utilization of Buprenorphine for Opioid Use Disorder After Implementation of the 2021 HHS Buprenorphine Practice Guidelines. https://aspe.hhs.gov/sites/default/files/documents/facbce1704035fded1034192d148304d/buprenorphine-practice-guideline-early-impacts.pdf.

[5] (2025). Fentanyl. https://www.cdc.gov/overdose-prevention/about/fentanyl.html.

[6] (2025). Naloxone. https://www.samhsa.gov/medication-assisted-treatment/medications-counseling-related-conditions/naloxone.

[7] PDAPS PDAPS (2025). Naloxone overdose prevention laws. http://pdaps.org/datasets/laws-regulating-administration-of-naloxone-1501695139..

[8] Abouk R, Pacula RL, Powell D (2019). Association between state laws facilitating pharmacy distribution of naloxone and risk of fatal overdose. JAMA Intern Med.

[9] Guy GP Jr, Haegerich TM, Evans ME, Losby JL, Young R, Jones CM (2019). Vital signs: pharmacy-based naloxone dispensing — United States, 2012-2018. MMWR Morb Mortal Wkly Rep.

[10] Mackey K, Veazie S, Anderson J, Bourne D, Peterson K (2020). Barriers and facilitators to the use of medications for opioid use disorder: a rapid review. J Gen Intern Med.

[11] Puzantian T, Gasper JJ (2018). Provision of naloxone without a prescription by California pharmacists 2 years after legislation implementation. JAMA.

[12] Banerjee S (2020). Optimizing the distribution of pharmacy-dispensed naloxone using spatial mapping techniques in rural areas. J Drug Issue.

[13] Abbas B, Marotta PL, Goddard-Eckrich D, Huang D, Schnaidt J, El-Bassel N, Gilbert L (2021). Socio-ecological and pharmacy-level factors associated with naloxone stocking at standing-order naloxone pharmacies in New York City. Drug Alcohol Depend.

[14] Carpenter DM, Dhamanaskar AK, Gallegos KL, Shepherd G, Mosley SL, Roberts CA (2019). Factors associated with how often community pharmacists offer and dispense naloxone. Res Social Adm Pharm.

[15] Thakur T, Frey M, Chewning B (2020). Pharmacist roles, training, and perceived barriers in naloxone dispensing: a systematic review. J Am Pharm Assoc (2003).

[16] Graves RL, Andreyeva E, Perrone J, Shofer FS, Merchant RM, Meisel ZF (2019). Naloxone availability and pharmacy staff knowledge of standing order for naloxone in Pennsylvania pharmacies. J Addict Med.

[17] Rudolph SE, Branham AR, Rhodes LA, Hayes HH Jr, Moose JS, Marciniak MW (2018). Identifying barriers to dispensing naloxone: a survey of community pharmacists in North Carolina. J Am Pharm Assoc (2003).

[18] Bakhireva LN, Bautista A, Cano S, Shrestha S, Bachyrycz AM, Cruz TH (2018). Barriers and facilitators to dispensing of intranasal naloxone by pharmacists. Subst Abus.

[19] Donovan E, Case P, Bratberg JP, Baird J, Burstein D, Walley AY, Green TC (2019). Beliefs associated with pharmacy-based naloxone: a qualitative study of pharmacy-based naloxone purchasers and people at risk for opioid overdose. J Urban Health.

[20] Tobin K, Clyde C, Davey-Rothwell M, Latkin C (2018). Awareness and access to naloxone necessary but not sufficient: examining gaps in the naloxone cascade. Int J Drug Policy.

[21] Hurt BR, Hussain A, Aledhaim A, Moayedi S, Schenkel SM, Kim HK (2020). Access and barriers to take-home naloxone use among emergency department patients with opioid misuse in Baltimore, Maryland, USA. Subst Use Misuse.

[22] Ko J, Chan E, Doroudgar S (2021). Patient perspectives of barriers to naloxone obtainment and use in a primary care, underserved setting: A qualitative study. Subst Abus.

[23] Bessen S, Metcalf SA, Saunders EC (2019). Barriers to naloxone use and acceptance among opioid users, first responders, and emergency department providers in New Hampshire, USA. Int J Drug Policy.

[24] (2025). Walgreens brand naloxone available online and in stores this month. https://investor.walgreensbootsalliance.com/news-releases/news-release-details/walgreens-brand-naloxone-available-online-and-stores-month.

[25] (2025). Naloxone distribution project. https://www.dhcs.ca.gov/individuals/Pages/Naloxone_Distribution_Project.aspx.

[26] Zhang A, Carrillo M, Liu R, Ballard SM, Reedy-Cooper A, Zgierska AE (2025). Vending machines for reducing harm associated with substance use and use disorders, and co-occurring conditions: a systematic review. Harm Reduct J.

[27] Becker WC, Sullivan LE, Tetrault JM, Desai RA, Fiellin DA (2008). Non-medical use, abuse and dependence on prescription opioids among U.S. adults: psychiatric, medical and substance use correlates. Drug Alcohol Depend.

[28] Heavey SC, Chang YP, Vest BM, Collins RL, Wieczorek W, Homish GG (2018). 'I have it just in case' - naloxone access and changes in opioid use behaviours. Int J Drug Policy.

[29] Bennett AS, Freeman R, Des Jarlais DC, Aronson ID (2020). Reasons people who use opioids do not accept or carry no-cost naloxone: qualitative interview study. JMIR Form Res.

[30] Marasinghe MG, Koehler KJ (2018). Statistical Data Analysis Using SAS.

[31] Schneider KE, Urquhart GJ, Rouhani S, Park JN, Morris M, Allen ST, Sherman SG (2021). Practical implications of naloxone knowledge among suburban people who use opioids. Harm Reduct J.

[32] Administration R-UF for F and D (2025). Naloxone Economic Review. https://reaganudall.org/sites/default/files/2023-03/Naloxone%20Report%20FINAL%203.8.23.pdf.

[33] Cariveau D, Fay AE, Baker D, Fagan EB, Wilson CG (2019). Evaluation of a pharmacist-led naloxone coprescribing program in primary care. J Am Pharm Assoc (2003).

[34] Evoy KE, Groff L, Hill LG, Godinez W, Gandhi R, Reveles KR (2020). Impact of student pharmacist-led naloxone academic detailing at community pharmacies in Texas. J Am Pharm Assoc (2003).

[35] Frank RG, Fry CE (2019). The impact of expanded Medicaid eligibility on access to naloxone. Addiction.

[36] Murphy SM, Morgan JR, Jeng PJ, Schackman BR (2019). Will converting naloxone to over-the-counter status increase pharmacy sales?. Health Serv Res.

[37] (2025). Naloxone is now available over-the-counter. Will it still be affordable?. https://healthlaw.org/naloxone-is-now-available-over-the-counter-will-it-still-be-affordable/.

